# Disruption of Interleukin-1β Autocrine Signaling Rescues Complex I Activity and Improves ROS Levels in Immortalized Epithelial Cells with Impaired Cystic Fibrosis Transmembrane Conductance Regulator (CFTR) Function

**DOI:** 10.1371/journal.pone.0099257

**Published:** 2014-06-05

**Authors:** Mariángeles Clauzure, Angel G. Valdivieso, María M. Massip Copiz, Gustavo Schulman, María Luz Teiber, Tomás A. Santa-Coloma

**Affiliations:** Laboratory of Cellular and Molecular Biology, Institute for Biomedical Research (BIOMED), School of Medical Sciences, Pontifical Catholic University of Argentina (UCA), and the National Scientific and Technical Research Council (CONICET), Buenos Aires, Argentina; University of Tübingen, Germany

## Abstract

Patients with cystic fibrosis (CF) have elevated concentration of cytokines in sputum and a general inflammatory condition. In addition, CF cells in culture produce diverse cytokines in excess, including IL-1β. We have previously shown that IL-1β, at low doses (∼30 pM), can stimulate the expression of CFTR in T84 colon carcinoma cells, through NF-κB signaling. However, at higher doses (>2.5 ng/ml, ∼150 pM), IL-1β inhibit CFTR mRNA expression. On the other hand, by using differential display, we found two genes with reduced expression in CF cells, corresponding to the mitochondrial proteins CISD1 and MTND4. The last is a key subunit for the activity of mitochondrial Complex I (mCx-I); accordingly, we later found a reduced mCx-I activity in CF cells. Here we found that IB3-1 cells (CF cells), cultured in serum-free media, secrete 323±5 pg/ml of IL-1β in 24 h vs 127±3 pg/ml for S9 cells (CFTR-corrected IB3-1 cells). Externally added IL-1β (5 ng/ml) reduces the mCx-I activity and increases the mitochondrial (MitoSOX probe) and cellular (DCFH-DA probe) ROS levels of S9 (CFTR-corrected IB3-1 CF cells) or Caco-2/pRSctrl cells (shRNA control cells) to values comparable to those of IB3-1 or Caco-2/pRS26 cells (shRNA specific for CFTR). Treatments of IB3-1 or Caco-2/pRS26 cells with either IL-1β blocking antibody, IL-1 receptor antagonist, IKK inhibitor III (NF-κB pathway) or SB203580 (p38 MAPK pathway), restored the mCx-I activity. In addition, in IB3-1 or Caco-2/pRS26 cells, IL-1β blocking antibody, IKK inhibitor III or SB203580 reduced the mitochondrial ROS levels by ∼50% and the cellular ROS levels near to basal values. The AP-1 inhibitors U0126 (MEK1/2) or SP600125 (JNK1/2/3 inhibitor) had no effects. The results suggest that in these cells IL-1β, through an autocrine effect, acts as a bridge connecting the CFTR with the mCx-I activity and the ROS levels.

## Introduction

Cystic fibrosis (CF) is an autosomal recessive disease caused by mutations in the cystic fibrosis transmembrane conductance regulator (*CFTR*) gene [1], [2], which encodes a chloride channel expressed at the apical surface of secretory epithelia [3], [4]. More than 1930 mutations have been identified in the *CFTR* gene [5]. The most common mutation, a deletion of three bases encoding a phenylalanine at position 508 (ΔF508), generates a misfolded CFTR protein. Consequently, the endoplasmic reticulum retains most of the CFTR, which then suffers proteasomal degradation [6], [7].

After the CFTR was cloned [1], [2] most studies were focused on non-genomic effects of CFTR. Little was known regarding its own gene regulation, except for effects of cAMP through CREB [8], and the enhanced mRNA degradation induced by TNF-α [9] or interferon-γ (but not interferon-α or β) [10]. Searching for other possible regulators of CFTR gene expression, we tested the effects of TGF-β1 and IL-1β. These particular proteins were selected because we had previously observed effects of TGF-β1 on other channels (calcium channels) [11], [12] and IL-1β usually had opposed effects to TGF-β1 [13]. Interestingly, we found that IL-1β, at doses up to 0.5–1.0 ng/ml (∼30–60 pM), was able to stimulate *CFTR* mRNA and protein expression, constituting the first extracellular upregulator known for CFTR [14], [15]. Although we did not further explore the effects of TGF-β1, later it was reported by Howe et al. that TGF-β1 down-modulates CFTR, an effect that was reverted by inhibitors of p38 MAPK, but not by inhibitors of JNK, ERK1/2 MAPK, or PI3K [16], [17]. Noteworthy, the response of *CFTR* to IL-1β was biphasic and, at doses over 2.5 ng/ml, IL-1β was inhibitory for the *CFTR* mRNA expression. In addition, the CFTR protein stimulation seen with lower IL-1β doses (∼0.5 ng/ml or 30 pM) was no longer observed in this second, inhibitory phase [15]. The first phase of CFTR response to IL-1β involved the NF-κB pathway [18]. The second phase has not been studied in detail yet, although preliminary data suggest that the *JNK→* c-Jun →*AP-1* pathway is involved [19]. Since the amount of IL-1β reported in sputum of CF patients (2.8–32 ng/ml) [20] is higher than the lowest *in vitro* inhibitory dose of 2.5 ng/ml, the IL-1β present in lungs should be enough to down-regulate CFTR, and it might had profound negative effects on the already reduced amounts of ΔF508 CFTR able to reach the cell membrane. Previously, Di Mango et al. had found elevated NF-κB activity and IL-8 production in CF cell lines [21]. It was later found that CFTR inhibition results on activation of NF-κB [22]–[24] and that several cytokines [25]–[31], including IL-1β [32], were upregulated in cultured CF cells. On the other hand, Velsor et al. found an altered glutathione balance and oxidative stress in CF cells [33], in agreement with earlier work of Burton Shapiro et al. [34](recently reviewed in [35]). Thus, excess of cytokines and a redox imbalance appear to be important characteristics of CF cells.

Soon after the CFTR was cloned it appeared evident certain lack of correlation between the CF genotype and the complex phenotype of the disease. We thought that this complex phenotype might be the consequence of a net of genes with altered expression due to the CFTR failure. Testing this hypothesis by using differential display, we found several CFTR-dependent genes [36]–[40]. Other laboratories found similar results by using microarrays [41]–[43]. One of the upregulated CFTR-dependent genes resulted to be *SRC/c-Src*. Since it was known that c-Src regulated the expression of several mucins, we thought that it might constitute a bridge between the CFTR failure and the overexpression of mucins in CF [38]. However, we know today that the CFTR → MUC pathway is more complex, since additional factors other than SRC/c-Src regulate mucins [44], [45]. Later, looking for differentially expressed genes that contrary to SRC showed a reduced expression in CF cells, we characterized two of them and, surprisingly, both resulted in genes codifying for mitochondrial proteins: *CISD1* (nuclear genome) [40] and *MTND4* (mitochondrial genome) [39]. Noteworthy, MTND4 had been reported as essential for the assembly and proper activity of mitochondrial Complex I (mCx-I) [46]. Due to the *MTND4* downregulation we observed in CF cells [39], we hypothesized that mCx-I activity should be also affected in CF cells or in cells with impaired CFTR function. In fact, we found later a reduced activity of mCx-I in CF cells or wt-CFTR treated with CFTR inhibitors or transfected with CFTR-shRNA [47]. Thus, a causal relationship between the CFTR and the mCx-I activities was demonstrated, although the mechanism was largely unknown [47]. These results were also in agreement with earlier reports of Burton Shapiro and colleagues [48]–[53], work that had been disregarded when the CFTR was cloned and found to be a chloride channel (reviewed in [35]).

In addition, pro-inflammatory cytokines, as IL-1β and TNF-α, are known to regulate mitochondrial function in human cardiomyocytes [54] and chondrocytes [55], inducing a reduced activity of mCx-I. Since it had been observed that IB3-1 cells in serum free culture media overexpressed IL-1β mRNA [32], we thought that the reduced activity of mCx-I found in CF cells might be mediated by increased levels of secreted IL-1β, somehow modulated by the CFTR activity. In agreement with this idea, here we show that IL-1β, through an autocrine effect, is responsible for the inhibition of the mCx-I and partially responsible for the increased reactive oxygen species (ROS) levels found in IB3-1 CF cells or in Caco-2/pRS26 cells (transfected with CFTR shRNA). These effects on mCx-I and ROS levels were reversed by using an anti-IL-1β monoclonal antibody able to block IL-1β signaling, interleukin-1 receptor antagonist (IL1RN) or by using pharmacological inhibitors of NF-κB activation (IKK inhibitor III/BMS-345541) or p38 MAPK (SB203580), but not by using inhibitors of JNK (SP600125) or MEK1/2 (U0126) kinases.

## Materials and Methods

### Reagents

Interleukin-1 beta (IL-1β) (Cat. No. I9401), IL-1 receptor antagonist (Cat. No. SRP3327), pepstatin, PMSF, leupeptin, dimethyl sulfoxide (DMSO, culture grade), NADH, dibutyryl-cAMP, lauryl-maltoside, IBMX, (−)-isoproterenol hydrochloride, protease inhibitor cocktail (Cat. No. P2714) and rotenone were purchased from Sigma-Aldrich (St. Louis, MO). Cytochrome c from equine heart (Cat. No. 250600), and IKK inhibitor III (BMS-345541) were from Calbiochem (San Diego, CA). MAPK1/p38 inhibitor (SB203580), MAPKK (MEK1/2) inhibitor (U0126) and JNK inhibitor (SP600125) were from Alomone Labs (Jerusalem, Israel). Bis-Tris, 6-aminohexanoic/ε-aminocaproic acid and nitroblue tetrazolium (NBT) were from Fluka (Sigma-Aldrich). NBT/BCIP mixture was from Promega (Madison, WI), and Coomassie Brilliant Blue G-250 was from Bio-Rad Laboratories (Hercules, CA). MitoSOX (Molecular Probes Cat. No. M36008), 2′,7′-dichlorofluorescein diacetate (DCFH-DA, Molecular Probes Cat. No. D399) and ROX (glycine conjugate of 5-carboxy-X-rhodamine, succinimidyl ester, Invitrogen Cat. No. 12223-012) were from Life Technologies Corporation (Carlsbad, CA). All other reagents were analytical grade. Antibodies: goat anti-mouse antibody coupled to alkaline phosphatase (goat polyclonal, sc-2008), goat anti-rabbit antibody coupled to alkaline phosphatase (goat polyclonal, sc-2007), mouse anti-ubiquinol-cytochrome c reductase core protein I (UQCRC1) (16D10 mAb, IgG_1_, sc-65238), mouse anti-NF-κB p65 (F-6 mAb, IgG_1_, sc-8008), mouse anti-IκB-α (H-4 mAb, IgG_1_, sc-1643), mouse anti p-p38 (D-8 mAb, IgM, sc-7973), mouse anti-p38α/β (A-12 mAb, IgG_1_, sc-7972), mouse anti-histone H1 (AE-4 mAb, IgG_2a_, sc-8030), and mouse anti-JNK2 (A-7 mAb IgG_1_, sc-271133) were from Santa Cruz Biotechnology Inc. (Santa Cruz, CA); mouse anti-IL-1β (mAb, IgG_1_, I3642; the antibody neutralizes the biological activity of recombinant human IL-1β) and rabbit anti-actin antibody (polyclonal, A2066) were from Sigma-Aldrich.

### Cell Cultures

IB3-1 cells (ATCC CRL-2777, a bronchial cell line derived from a cystic fibrosis patient with a ΔF508/W1282X CFTR genotype) [56] and S9 cells (ATCC CRL-2778, which are IB3-1 cells transduced with an adeno-associated viral vector to stably express wt-CFTR) [57] were purchased from ATCC (www.atcc.org) (these cell lines are no longer provided by ATCC; they are kept now at the John Hopkins University Cell Center). Caco-2/pRS26 cells that stably express a shRNA plasmid directed against CFTR and Caco-2/pRSctrl control cells (HuSH 29-mer shRNA Non-Effective Expression Plasmid against GFP as negative control; OriGene Rockville, MD) were cultured as previously described [47] adding 1 µg/ml puromycin. All cells were cultured in DMEM/F12 (Life Technologies, GIBCO BRL products, Rockville, MD) supplemented with 5% FBS (Fetal Bovine Serum)(PPA GmbH, Austria), 100 units/ml penicillin, 100 µg/ml streptomycin, and 0.25 µg/ml amphotericin B (Life Technologies, GIBCO BRL, Rockville, MD). Cells were seeded at a density of 20,000 cells/cm^2^ (p150 dishes have ∼140 cm^2^) and cultured for 24 h in 15 ml (0.107 ml/cm^2^) DMEM-F12 plus 5% FBS, at 37°C in a humidified air atmosphere containing 5% CO_2_. Before treatments, cells were cultured 24 hours in serum-free medium. Then, the serum-free media was replaced and the cells were cultured for additional 24 h (S9 and IB3-1 cells in the presence of a CFTR-stimulation cocktail: 200 µM dibutyryl-cAMP, 200 µM IBMX and 20 µM isoproterenol). The different treatments (IL-1β, anti-IL1β, IL-1 receptor antagonist and inhibitors) were performed in this second 24 h period, in serum free media. The media volume used was always 0.107 ml/cm^2^.

### Reverse Transcription and Quantitative Real-time PCR (qRT-PCR) for IL-1β

The IB3-1 and S9 cells were cultured 72 h as above indicated, and total RNA isolated by using a guanidinium thiocyanate-phenol-chloroform extraction solution [58]. The quality of RNA was checked by electrophoresis in denaturing formaldehyde agarose gels [59], and measuring the ratios A260/A230 (greater than 2) and A260/A280 nm (from 1.7 to 2.0). Total RNA samples (1.5 µg) were transcribed by using M-MLV Reverse Transcriptase (Promega) and Oligo-dT_12_, according to the manufacturer’s instructions (100 U of RT/µg of RNA). Quantitative real-time RT-PCRs (qRT-PCR) were performed by using an ABI 7500 real-time PCR system (Applied Biosystems Inc., Foster City, CA); the ΔΔCt method was used for comparative quantification. *TBP* (Tata Box Binding Protein) was used as an internal control. Primer sequences for PCR were as follows: *TBP*, 5′-TGCACAGGAGCCAAGAG TGAA-3′ (forward) and 5′-CACATCACAGCTCCCCACCA-3′ (reverse); IL*-1β*, 5′-ACAGATGAAGTGCTCCTTCCA-3′ (forward) and 5′-GTCGGAGATTCGTAGCTGGAT-3′ (reverse). The cDNA samples (10 µl of a 1∶10 of cDNA from reverse-transcribed RNA) were added to 25 µl of PCR reaction mixture containing a final concentration of 2.5 mM MgCl_2_, 0.4 mM deoxynucleotide triphosphates, 1 U of Go Taq DNA polymerase (Promega), 0.1 X EvaGreen (Biotium, Hayward, CA), 50 nM ROX as reference dye, and 0.2 µM of each primer. The qRT-PCR conditions were as follows: initial denaturation at 95°C for 10 min, followed by 40 cycles at 95°C for 30 s, 62°C for 30 s and 72°C for 30 s. Fluorescence signal was acquired at the elongation step, at the end of each cycle. qRT-PCR reactions were carried out in triplicates (intra- and inter-assays by triplicate). The final quantification values were obtained as the mean of the Relative Quantification (RQ) for each biological triplicate (n = 3).

### Secretion of IL-1β

Secreted IL-1β was measured in conditioned media from IB3-1 cells, S9 cells, Caco-2/pRS26 cells and Caco-2/pRSctrl control cells. Each cell lines was cultured 72 h as above indicated in five p150 dishes (the last 24 h in 10 ml of serum free media, except for S9 and IB3-1 cells which also have CFTR stimulation cocktail). The media were collected and concentrated by centrifugation at 3500×g for 30 min at 4°C; by using the Amicon Ultra-15 centrifugal filter units (10.000 kDa cut-off, EMD Millipore, Billerica, MA). Protein concentration was determined by Lowry [60]. Then, an immune-dot blotting procedure previously described by Sun et al. [61] was applied to determine the IL-1β levels in the concentrated medium, with some modifications. Briefly, the concentrated samples (150 µL derived from 50 ml of conditioned media) and different amounts of recombinant IL-1β (0, 0.3125, 0.625, 0.125, 2.5 and 5 ng) were slowly filtered through a nitrocellulose membrane by using the Easy-Titer ELIFA System (Thermo Scientific Pierce, Rockford, IL) [62]–[64]. The membranes were then blocked with 5% BSA in TBS (25 mM Tris, 150 mM NaCl, pH 7.0), washed with TBS, and incubated with a monoclonal antibody raised against IL-1β (Sigma I3642, dilution 1∶1000 in TBS plus Tween-20, 0.05% v/v). After 3 h incubation with the primary antibody, the membranes were washed three times with TBS plus Tween-20 (0.05% v/v) for 5 min. Then, the membranes were incubated with a secondary goat IgG anti-mouse antibody coupled to alkaline phosphatase (Santa Cruz Biotechnology, sc2008, dilution 1∶1000), washed three times with TBS plus Tween-20 (0.05% v/v) for 5 min and developed with the substrates NBT-BCIP, following the manufacturer’s instructions (Promega). The membranes were scanned (HP ScanJet G3110 scanner) and the signal intensities quantified by densitometry by using the NIH Image software, Java version (ImageJ, rsbweb.nih.gov). Finally, densitometry values were used to calculate the IL-1β amounts through interpolation into the standard curve of IL-1β (0–5 ng/ml), and were expressed as concentration values (pg/ml and pM).

### Mitochondria Isolation

Mitochondria were isolated by using differential centrifugation, according to Majander et al. [65], with minor modifications [47]. Briefly, cells were incubated 72 h as above indicated (see Cell cultures section). After treatment, cells were maintained at 0–4°C (over ice/water), washed with cold PBS, scrapped in the presence of 1 ml PBS with protease inhibitors (10 µM pepstatin, 10 µM leupeptin, 100 µM PMSF, 1 mM EDTA) and centrifuged at 600×g, 5 min at 4°C. The pellet was resuspended in isolation buffer (0.25 M sucrose, 25 mM MOPS, pH 7.4, plus the same protease inhibitors added to PBS), diluted to 250 mg/ml, and the cells were permeabilized by adding 0.12% w/v digitonin for 30 s on ice. The sample was diluted in three volumes of isolation buffer and centrifuged at 10,000×g for 20 min at 4°C. The resultant pellet was resuspended in 500 µl of isolation buffer and centrifuged at 800×g for 10 min at 4°C. The supernatant was centrifuged at 10,000×g for 20 min at 4°C, to collect the mitochondria. The mitochondrial pellet was resuspended in 10–20 µl of BN-sample buffer A (1 M aminocaproic acid, 50 mM Bis-Tris-HCl, 10 µM pepstatin, 10 µM leupeptin, 100 µM PMSF, 1 mM EDTA, pH 7.0). Mitochondrial protein concentration was measured by the Lowry’s method by using aliquots of mitochondrial extract incubated with 0.1 N NaOH for 30 min at 37°C, to dissolve mitochondrial membranes [60].

### Blue Native PAGE (BN-PAGE)

The BN-PAGEs were performed according to Schägger et al. [66], with minor modifications [47]. Mitochondrial protein samples were resuspended in BN-sample buffer A (see above) to a final protein concentration of 3 µg/µl, and then solubilized adding lauryl maltoside (0.6% w/v, final concentration). The mixture was then centrifuged at 20,000×g for 30 min at 4°C. Before loading the gel, BN-sample buffer B containing Coomassie Brilliant Blue G-250 (CBB G-250) (1 M aminocaproic acid, 20% glycerol, 50 mM Bis-Tris-HCl, 5% w/v CBB G-250, pH 7.0) was added to the samples at a final ratio 1∶14 v/v. Samples (80 µg of protein) were electrophoresed in a 5–13% BN-PAGE gradient gel containing a 4% stacking gel. Electrophoresis was carried out at 80 V, at 4°C, until protein samples migrated into the stacking gel. Then, the voltage was set to 200 V with a current limited to 15 mA (voltage 200→400 volts), at 4°C. Once the trace dye had migrated half-way into the separation gel, the cathode (−) buffer (50 mM Tricine, 15 mM Bis-Tris-HCl, 0.02% CBB G-250, pH 7.0) was replaced with the same buffer without CBB G-250, in order to reduce the background of gels [47].

### Measurement of Mitochondrial Complex I (mCx-I) in-gel Activity (IGA) and Western Blotting of Mitochondrial Proteins

To measure the mCx-I (IGA) at the end of the run, the upper section of the separation gel (around 2 cm), containing the mCx-I, was incubated for 20–40 min in development buffer (0.1 M Tris-HCl, 0.14 mM NADH, 1.22 mM NBT, pH 7.4). To stop the reaction and distain the CBB background, a fixing solution (45% methanol: 10% acetic acid in water) was used. In parallel, the inferior section of the gel was transferred to a PVDF membrane using a transfer buffer without methanol (39 mM glycine, 48 mM Tris-base, 0.037% sodium dodecyl sulfate (SDS), pH 8.3) for 2.5 h at 100 V (constant voltage). The membranes were blocked with 5% BSA 1 h in TBS buffer and incubated with a monoclonal antibody raised against the ubiquinol-cytochrome c reductase core protein I (UQCRC1), a subunit of the mitochondrial complex III (mCx-III, EC 1.10.2.2) (mAb sc-65238 from Santa Cruz Biotechnology; dilution 1∶500 in TBS plus Tween-20, 0.05% v/v). After 3 h incubation with the primary antibody, the membranes were washed three times with TBS plus Tween-20 (0.05% v/v) for 5 min. Then, the membranes were incubated with a secondary goat IgG anti-mouse antibody coupled to alkaline phosphatase (mAb sc-2008 from Santa Cruz Biotechnology; dilution 1∶1000 in TBS plus Tween-20, 0.05% v/v), washed three times with TBS plus Tween-20 (0.05% v/v) for 5 min and developed with the substrates NBT-BCIP following the manufacturer’s instructions (Promega). Membranes and gels were scanned and the signal intensities quantified by using the Image J software. The mCx-I activity was referred to the UQCRC1 values, used as internal controls. This subunit was used as internal standard since its concentration remained constant with the different treatments.

### Spectrophotometric Assay of Mitochondrial NADH-cytochrome C Reductase Activity

To measure the mCx-I activity using cytochrome c as an natural electron acceptor instead of NBT (used for BN-PAGE), we measured the NADH-cytochrome c reductase activity (mCx-I plus mCx-III) spectrophotometrically, in the presence/absence of rotenone (10 µM) (Sigma R8875), an specific inhibitor of mCx-I, as it was previously described by others [67], [68], with minor modifications. Briefly, mitochondrial preparations were subjected to three freeze-thaw cycles to make them permeable to substrates. To measure the mCx-I activity, mitochondria (equivalent to 100 µg of proteins; to obtain consistent results it is important not to use less than 100 µg) were resuspended in a buffer solution (100 mM H_2_KPO_4_/HK_2_PO_4_, 0.5 mM KCN, 200 µM NADH, 25 µM cytochrome c, pH 7.4) and the reduction of cytochrome c was recorded as absorbance/min at 550 nm, for 2 min at and 30°C. To calculate the enzyme activity, inhibition of NADH cytochrome c reductase activity by rotenone (10 µM) was measured in parallel for each sample after 5 min of preincubation with the inhibitor, and the remaining values (insensitive to rotenone) were subtracted from the total activity. Then, the results were expressed as percentages, considering the activity in control cells as 100%. The measurement of mCx-I and mCx-III activities in a chain reaction (NADH→mCx-I→mCx-III→cytochrome c) is a good indicative that the respiratory chain complexes remain intact after mitochondrial isolation and also provides a way to use a natural electron acceptor as cytochrome c instead of NBT, reinforcing the results of BN-PAGEs.

### Subcellular Fractionation to Determine NF-κB and p38 Activities

A subcellular fractionation was performed as previously described by Wier et al. [69]. Briefly, cells were incubated as above indicated. Then, cells were washed twice with cold PBS, scraped with 1 ml cold PBS with protease inhibitors (10 µM pepstatin, 10 µM leupeptin, 100 µM PMSF, 1 mM EDTA), and centrifuged at 600×g for 10 min at 4°C. The cell pellet was resuspended in 500 µl of buffer containing 10 mM HEPES, 1.5 mM MgCl_2_, 10 mM KCl, 0.5 mM fresh dithiothreitol (DTT), 0.4% NP-40, and protease inhibitor cocktail (Sigma-Aldrich P2714), pH 7.9, for 5 min at 4°C. The cell lysate/nuclear suspensions were prepared by centrifugation at 500×g for 3 min at 4°C, and supernatants were collected as cytosolic fractions. Then, the pellets were resuspended in nuclear extraction buffer (20 mM HEPES, 0.4 M NaCl, 1.5 mM MgCl_2_, 0.2 mM EDTA, 25% glycerol, 0.5 mM DTT and protease inhibitor cocktail, pH 7.9). The samples were kept on ice for 10 min, and centrifuged at 14,000×g for 10 min at 4°C. The supernatant was used as the nuclear extract and stored at −80°C before use. The protein concentration was determined by Lowry [60].

### Western Blot Analysis of NF-κB and p38

Western blots (WB) were performed as previously described [18]. Briefly, nuclear and cytosolic extracts (30–50 µg of proteins) were separated on a denaturing SDS-PAGE (12%) and transferred to nitrocellulose membranes. Cytosolic proteins were analyzed for p-p38, p38, IκB-α and actin, and nuclear proteins were analyzed for NF-κB p65 subunit and histone H1. Membranes were blocked with BSA 5% in TBS 1 h and then incubated with primary monoclonal antibodies against NF-κB p65 subunit, IκB-α and p-p38 (Santa Cruz Biotechnology; sc-8008, sc-1643, sc-7973, respectively; dilutions 1∶500 in TBS plus Tween-20, 0.05% v/v) for 3 h. The membranes were washed three times with TBS plus Tween-20 (0.05% v/v) for 5 min and incubated for 1 h with goat IgG anti-mouse antibody coupled to alkaline phosphatase (mAb sc-2008 from Santa Cruz Biotechnology; dilution 1∶1000 in TBS plus Tween-20, 0.05% v/v), washed three times with TBS plus Tween-20 (0.05% v/v) for 5 min and developed with the substrates NBT-BCIP following the manufacturer’s instructions (Promega). As internal controls, membranes were reincubated with anti-actin antibody (Sigma-Aldrich, A2066, dilution 1∶1000), anti-p38 (Santa Cruz Biotechnology; sc-7972, dilution 1∶500) or anti-histone H1 (Ab AE-4, sc-8030, Santa Cruz Biotechnology, dilution 1∶500), washed three times as above indicated and then incubated for 1 h with goat IgG anti-mouse antibody coupled to alkaline phosphatase (mAb sc-2008 from Santa Cruz Biotechnology; dilution 1∶1000 in TBS plus Tween-20, 0.05% v/v) or goat IgG anti-rabbit antibody coupled to alkaline phosphatase (mAb sc-2007 from Santa Cruz Biotechnology; dilution 1∶1000 in TBS plus Tween-20, 0.05% v/v) as secondary Ab for actin. Membranes were developed as mentioned before and scanned. The signal intensities were quantified by using the Image J software.

### Measurement of Mitochondrial and Cellular ROS Levels

Mitochondrial and cellular ROS levels were measured by using fluorescent probes in 96 well black plates (Greiner Bio-One, Germany; 655090). The cells were cultured 72 h as above indicated and treated the last 24 h with IL-1β, anti-IL-1β blocking monoclonal antibodies or inhibitors of JNK (SP600125), MAPK1/p38 (SB203580), MAPKK/MEK1/2 (U0126), and IKK (IKK inhibitor III). To measure mitochondrial ROS levels, at the end of incubation, the DMEM-F12 medium was changed to Hank’s medium (136.9 mM NaCl, 5.4 mM KCl, 1.3 mM CaCl_2_, 3.7 mM NaH_2_PO_4_, 0.4 mM KH_2_PO_4_, 4.2 mM NaHCO_3_, 0.7 mM MgSO_4_, 5.5 mM D-glucose and 10 mM HEPES) containing 5 µM of MitoSOX [70] (stock prepared as 5 mM solution in DMSO) and incubated at 37°C in the 5% CO_2_/air incubator for 10 min. Cellular ROS levels were measured by using the fluorescent probe DCFH-DA in Hank’s medium containing 10 µM of the fluorescent probe (stock prepared as 20 mM solution in DMSO) and incubated at 37°C in the 5% CO_2_/air incubator for 40 min. Then, cells were washed with 0.2 ml of Hank’s buffer three times and the fluorescence was measured in a fluorescence plate reader (NOVOstar BMG LABTECH GmbH Ortenberg, Germany) with incubation at 37°C. Filters were Ex = 510±10 nm, Em = 580±10 nm for MitoSOX and Ex = 510±10 nm, Em = 540±10 nm for DCFH-DA, and readings were performed by using 10 cycles (3 flashes per well and cycle, excitation and measurements done from the bottom of the plate).

### Statistics

Unless otherwise indicated, the assays were performed at least by duplicates and the experiments were repeated at least three times. The results were expressed as the media obtained from the different independent experiments (interassay comparisons). One-way ANOVA and the Tukey’s test were applied to calculate significant differences among samples (α = 0.05). All values are indicated as mean ± SE (n). *indicates significant differences (p<0.05).

## Results

### Expression of Interleukin-1 Beta in IB3-1and S9 Cells

**Figure 1:**
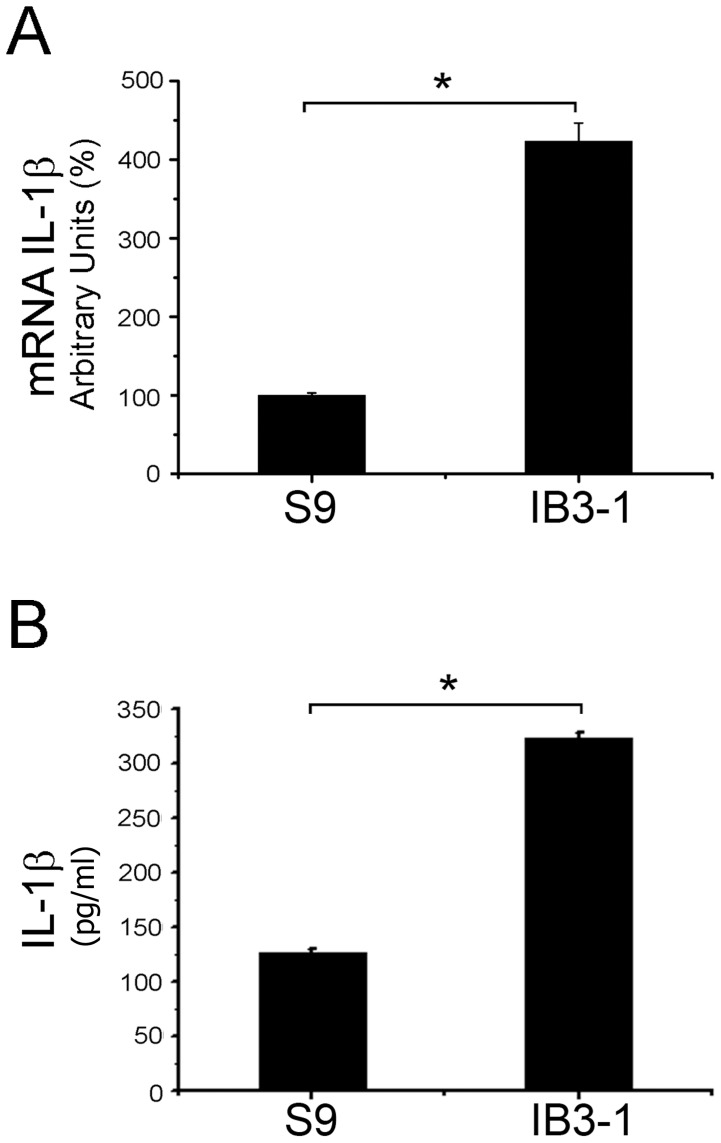
Interleukin-1 beta expression in IB3-1 and S9 cells. IB3-1 and S9 cells were preincubated for 24 h in serum-free DMEM/F12 plus 24 h in the presence of a CFTR-stimulating cocktail and the cellular IL-1β mRNA and protein levels were determined. A: Quantitative real-time RT-PCR of IL-1β mRNA expression levels in S9 e IB3-1 cells. B: Immune-dot blotting quantification of the IL-1β present in culture media. The results were expressed as pg/ml in the conditioned media, after 24 h of culture. Measurements were performed in duplicate and data are expressed as mean ± SE of three independent experiments (n = 3). *indicates p<0.05.

In order to test whether in our cultured conditions (serum starvation plus CFTR stimulation) IB3-1 cells overexpress IL-1β compared to S9 cells (which are IB3-1 CF cells rescued by ectopic expression of wt-CFTR), we measured the relative levels of IL-1β mRNA and protein. As shown in Figure 1A, expression of IL-1β mRNA was significantly increased (>400%; p<0.05) in IB3-1 cells compared to S9 cells. These results were in agreement with the values reported by Bartling et al. for the same cell lines [32]. In addition, a significant although less pronounced increment was observed for the secreted protein, accumulated during 24 h of culture: 127±3 pg/ml (n = 2)(∼7.3 pM) for S9 cells and 323±5 pg/ml (n = 2)(∼18.5 pM) for IB3-1 cells (2.5 fold increase, p>0.05), as shown in Figure 1B. The secretion rate was 2.7 fmol/10^6^cell/h or 15.7 fg/ml/mg/h (mg of total proteins in the culture media) for IB3-1 and 1.1 fmol/10^6^cell/h or 6.5 fg/ml/mg/h for S9 cells (assuming a constant rate over the 24 h period).

### Exogenous IL-1β Affects the Mitochondrial Complex I (mCx-I) Activity

**Figure 2:**
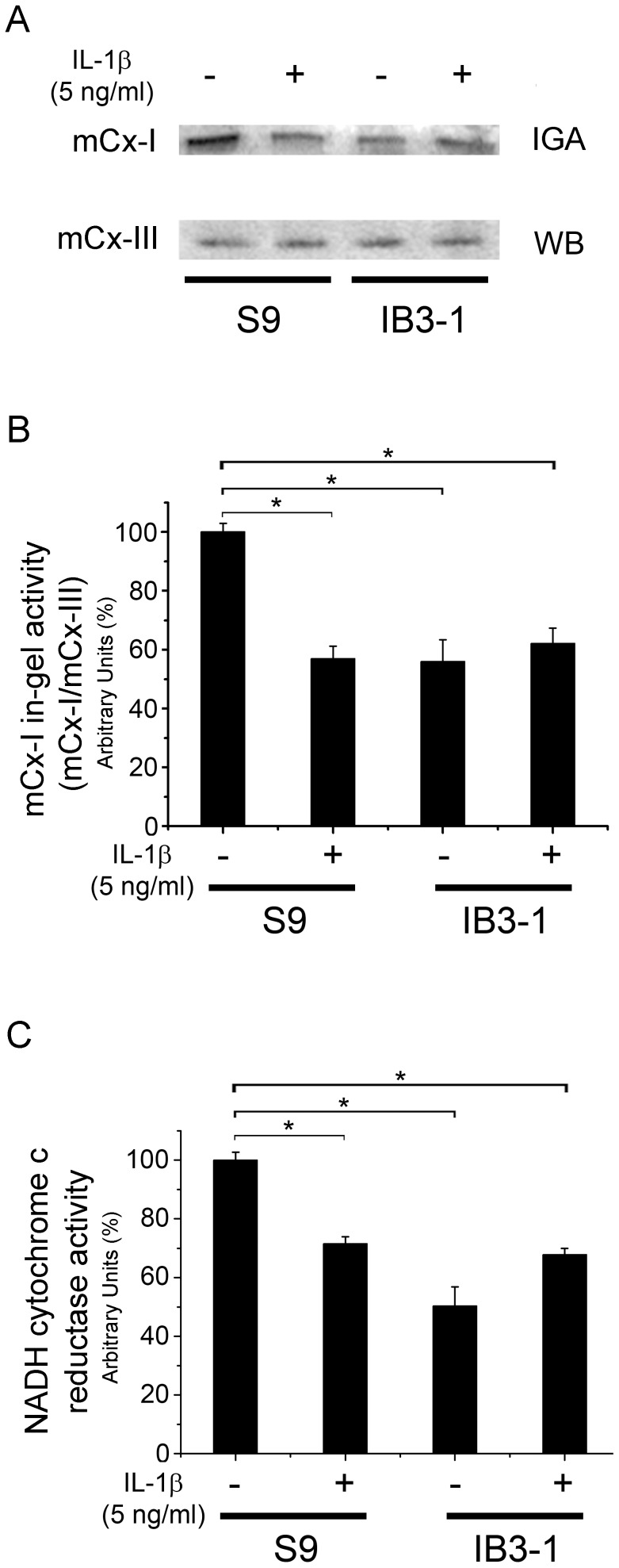
Effect of IL-1β on mitochondrial Complex I activity (mCx-I). IB3-1 and S9 cells pre-incubated in serum free media for 24 h were incubated for additional 24 h (plus CFTR-stimulating cocktail) in the presence or absence of IL-1β (5 ng/ml) and the mCx-I activity was determined. A: mCx-I in-gel activity (IGA) of treated and untreated S9 and IB3-1 cells; mCx-III correspond to the WB for the UQCRC1 subunit of the mitochondrial complex III used as internal standard. B: Densitometric quantification and statistical analysis of the results shown in panel A (for n = 3); IGA of mCx-I was calculated as the ratio mCx-I IGA/mCx-III WB. C: Spectrophotometric measurements of mitochondrial NADH-cytochrome c reductase activity for the same experiments shown in panel A and B. Results were expressed as percentage (%) relative to S9 control values. Measurements were performed in triplicate and data were expressed as mean ± SE of three independent experiments (n = 3). *indicates p<0.05 compared with basal S9 cells.

We have previously found that the mCx-I activity was reduced approximately 50% in CF cells or in cells with impaired CFTR-function (inhibitors, shRNA), cultured for 24 h in serum-free media [47]. In the presence of FBS, differences in mCx-I activity were not observed (data not shown). Now, to study the possible role of IL-1β in the reduced mCx-I activity of CF cells, we first tested whether the addition of exogenous IL-1β might reduce the mCx-I activity of S9 and IB3-1 cells. Both cell lines were incubated with 5 ng/ml of IL-1β for 24 h in serum-free medium (in presence of CFTR-stimulating cocktail). As shown in Figure 2, the mCx-I in-gel activity (IGA) of S9 cells was significantly reduced (p<0.05), as compared to untreated control cells. However, no significant differences (p>0.05) were observed between IL-1β treated and untreated IB3-1 cells. Similar results were found when the mitochondrial NADH-cytochrome c reductase activity was measured spectrophotometrically, as shown in Figure 2C. It should be pointed-out however, that the reduction in the NADH-cytochrome c reductase activity of IB3-1 cells compared to S9 cells was variable, in a range between 20% and 50%. To avoid this problem, at least 100 µg of total protein should be used for the assays. Altogether, these results confirm that IB3-1 cells, have a reduced mCx-I activity, and demonstrate that exogenous added IL-1β is able to reduce the mCx-I in S9 cells (which are CFTR-corrected IB3-1 cells). The results were in agreement with previous reports showing that IL-1β impaired the mCx-I activity in cardiomyocytes [54] and chondrocytes [71].

The lack of response to exogenous IL-1β (5 ng/ml) obtained with IB3-1 cells suggests that these cells might have already a saturated IL-1β signaling, probably due to the presence of secreted IL-1β in abundance (with a saturated autocrine signaling). In this regard, the IL-1β concentration in the conditioned media of IB3-1 cells after 24 h was 323 pg/ml (18.5 pM), which is three times less than the concentration required for maximal mCx-I inhibition in S9 cells (∼57 pM or 1 ng/ml) [72]. However, the actual concentration of IL-1β in the cell surrounding volume should be several times higher than in the total volume of the conditioned media, as it was similarly observed for ATP secretion and signaling [73], [74].

### Recovery of mCx-I Activity by Incubation with Anti-IL-1β Blocking mAb or IL-1 Receptor Antagonist

**Figure 3:**
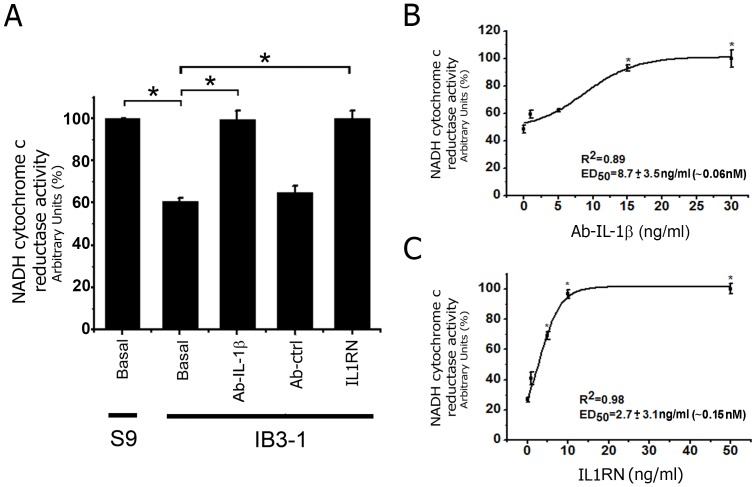
Recovery of mCx-I activity by incubation with anti-IL-1β blocking antibody or with IL-1β receptor antagonist (IL1RN). S9 and IB3-1 cells preincubated for 24 h in serum free media were additionally incubated for 24 h (in the presence of a CFTR-stimulating cocktail) with anti-IL-1β blocking antibody or IL-1 receptor antagonist. The mCx-I activity was determined by using spectrophotometric measurements of the mitochondrial NADH-cytochrome c reductase activity. A: IB3-1 cells were treated with anti-IL-1β blocking antibody (30 ng/ml) or IL-1 receptor antagonist (10 ng/ml). The Figure shows the activity expressed as percentage (%), considering the average activity of S9 cells as 100%. Ab-Ctrl: anti-JNK2 monoclonal antibody as negative control (30 ng/ml). B: Spectrophotometric measurements of mitochondrial NADH-cytochrome c reductase activity of IB3-1 cells treated with increasing concentrations of anti-IL-1β blocking antibody (0, 1, 5, 15 and 30 ng/ml). C: Spectrophotometric measurements of mitochondrial NADH-cytochrome c reductase activity of IB3-1 cells treated with increasing concentrations of IL-1 receptor antagonist (0, 1, 5, 10 and 50 ng/ml). Measurements were performed in triplicate and data were expressed as mean ± SE of three independent experiments (n = 3). *indicates p<0.05 compared with basal IB3-1 cells.

To confirm the presence of an autocrine IL-1β secretion in IB3-1 cells and its possible effect on the mitochondrial mCx-I activity, we used an anti-IL-1β monoclonal antibody (IgG_1_ isotype) or the IL-1 receptor antagonist (IL1RN) to block the IL-1β binding to its receptor [75], [76]. As shown in Figure 3, treatment of IB3-1 cells with increasing concentrations of blocking antibody (cells incubated 24 h in serum-free medium in presence of CFTR-stimulating cocktail plus different Ab concentrations) or IL1RN results in a significant (p<0.05) increment of the mitochondrial NADH-cytochrome c reductase activity sensitive to rotenone. Dose-response curves for the IL-1β blocking Ab and the IL1RN are shown in Figures 3B and 3C (IL-1β blocking Ab ED_50_ = 8.76±3.59 ng/ml or ∼0.06 nM; IL1N ED_50_ = 2.70±3.11 ng/ml or ∼0.15 nM). These results are in agreement with previous observations of Verhaeghe et al. showing the presence of an autocrine IL-1β loop in cultured epithelial CFT-2 and 16HBE CF cells, which could be disrupted by using anti-IL-1β blocking antibodies [77]. Noteworthy, the IL-1β blocking Ab (5 ng/ml and over) and the IL-1RN were able to rescue the mCx-I activity of IB3-1 cells to values very close to those observed in S9 cells as a reference. In other words, a complete recovery of the mCx-I activity seems to occur in the presence of the blocking Ab or IL1RN. By the contrary, control antibodies anti-JNK (IgG_1_) (Fig. 3A) or anti-Histone (IgG_2a_, data not shown) showed no effects over basal mCx-I activity. These results reinforce the idea that secreted IL-1β (Fig. 1) is acting as an autocrine factor affecting the mCx-I activity in CF cells.

### Effects of IL-1β Pathway Inhibitors on the Reduced mCx-I Activity of IB3-1 CF Cells

To reinforce the idea that an autocrine IL-1β signaling is responsible for the inhibition of mCx-I activity in IB3-1 cells, in addition to the anti-IL-1β blocking Ab we used four pharmacological inhibitors of key enzymes involved in IL-1β signaling. The results obtained are described below.

### Effects of MEK1/2 Inhibition on mCx-I Activity

**Figure 4:**
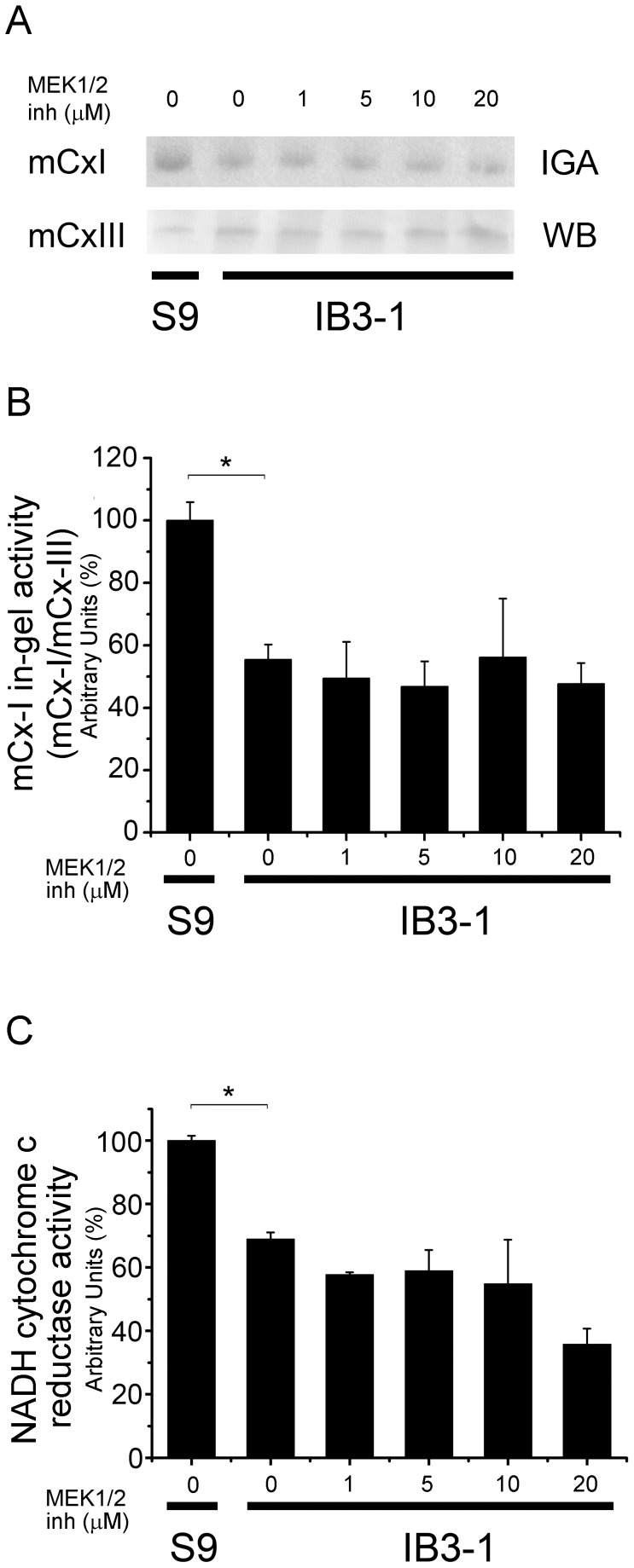
MEK1/2 inhibition. After 24-1 cells were incubated for 24 h with increasing concentrations of the MAPK/AP-1 pathway inhibitor U0126 (1, 5, 10 and 20 µM) and the mCx-I activity was measured by using BN-PAGE and spectrophotometry. A: mCx-I in-gel activity (IGA) and mCx-III Western blotting (WB). B: Densitometric quantification and statistical analysis of the results shown in panel A, calculated from three independent experiments (n = 3). IGA of mCx-I was calculated as the ratio mCx-I (IGA)/mCx-III (WB). C: Spectrophotometric measurements of the mitochondrial NADH-cytochrome c reductase activity in the same experiments of panel A, expressed as percentage (%) relative to S9 control values. Measurements were performed in triplicate and data were expressed as mean ± SE of three independent experiments (n = 3). *indicates p<0.05 compared with basal IB3-1 cells.

MEK1/2 kinases belong to a p42/p44 branch of the IL-1β signaling pathway. This branch, made by RAS, RAF, MEK1/2 and ERK1/2, forms a bridge between the IL-1β receptor I signaling and the AP-1 transcription factor. In human and mouse epidermis, the single disruption of MEK1 or MEK2 have no effect, whereas double mutants abolish ERK1/2 phosphorylation, leading to hypo-proliferation and apoptosis, effects that can be rescued overexpressing ERK1/2 [78]. In order to inhibit MEK1/2 kinases, we used the pharmacological inhibitor U0126. It has been shown to inhibit MEK1/2 kinases with IC_50_ = 0.072±0.02 µM for MEK1 and IC_50_ = 0.058±0.02 µM for MEK2 [79]. The AP-1 activity was also inhibited in COS-7 cells incubated for 24 h in the presence of U0126 with an IC_50_ = 0.96±0.16 µM for [79]. Accordingly, we used different doses of inhibitor starting at 1 µM to assure inhibition of AP-1 signaling. As shown in Figure 4, incubation of IB3-1 cells with up to 20 µM of U0126 did not resulted in any improvement of the low mCx-I activity of these cells. By the contrary, U0126 shows a tendency to reduce the mCx-I activity of IB3-1 cells (more evident in Fig. 4C than in Fig. 4B). Since the IC_50_ for AP-1 ∼ 1 µM (and less for MEK1/2) [79], these results suggest that the AP-1 pathway (including MEK1/2 kinases) is not be involved in the autocrine effects of IL-1β over the mCx-I activity.

### Effects of JNK Inhibition on mCx-I Activity

**Figure 5:**
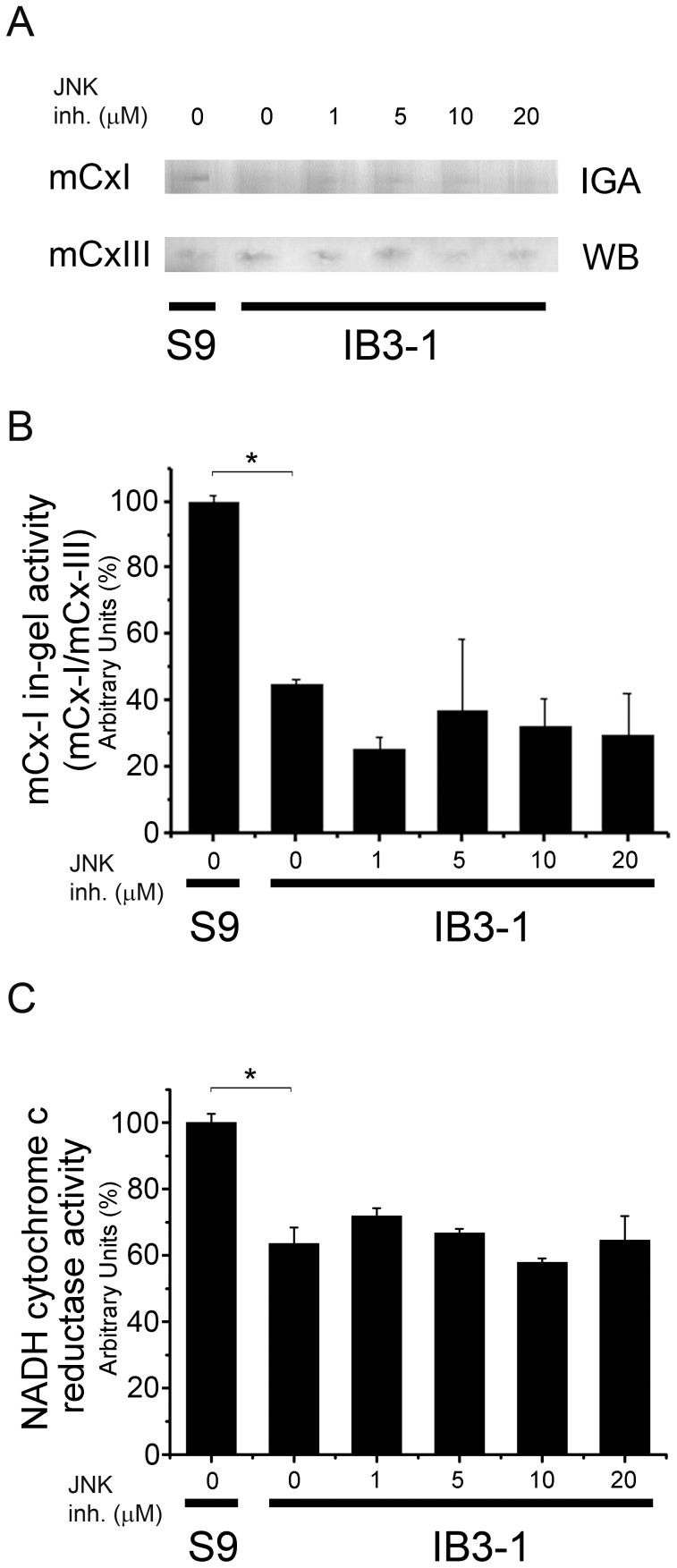
JNK inhibition. IB3-1 cells preincubated for 24 h in serum free media were additionally incubated for 24 h with increasing concentrations of the JNK inhibitor SP600125 (1, 5, 10 and 20 µM) and the mCx-I activity was measured by using BN-PAGE and spectrophotometry. A: mCx-I in-gel activity (IGA) and mCx-III (WB). B: Densitometric quantification and statistical analysis of the results shown in panel A. IGA of mCx-I was calculated as the ratio mCx-I (IGA)/mCx-III (WB). C: Spectrophotometric measurements of the mitochondrial NADH-cytochrome c reductase activity in samples obtained from the same experiments of panel A, expressed as percentage (%) relative to S9 control values. Measurements were performed in triplicate and data were expressed as mean ± SE of three independent experiments (n = 3). *indicates p<0.05 compared with basal IB3-1 cells.

Jun N-terminal kinases (JNKs) are stress kinases that can be activated by inflammatory cytokines (including IL-1β), bacterial endotoxin, osmotic shock, UV radiation and hypoxia [80]. SP600125 has been shown to inhibit Jun N-terminal kinases (JNKs) and other kinases (IC_50_ = 40 nM for JNK1 and JNK2, 90 nM for JNK3 and 1.0–5.1 µM for p56Lck, MKK3/4/6/7, PKB/AKT and PKCα [80]). In Jurkat cells, SP600125 blocks c-Jun phosphorylation with an IC_50_ = 5–10 µM [80]. Therefore, we used this inhibitor at concentrations ranging (1–20 µM). As shown in Figure 5, increased concentrations of SP600125 were not able to recover the reduced mCx-I activity of IB3-1 cells. These results, as those obtained with U0126, also suggest that AP-1 signaling is not involved in the autocrine effects of IL-1β over the mCx-I activity.

### Effects of p38 Inhibition on mCx-I Activity

The p38 mitogen-activated protein kinases are also activated by different cellular stresses, bacterial lipopolysaccharide (LPS) and inflammatory cytokines, including IL-1β [81]. These kinases are also involved in the regulation of pro-inflammatory cytokine expression [82]. There are four p38 MAPKs identified so far: MAPK14/p38-α/SAPK2A, MAPK11/p38-β/SAPK2B, MAPK12/p38-γ/SAPK3 and MAPK13/p38-δ/SAPK4. SB203580 has been used to inhibit MAPK14/p38-α and MAPK11/p38-β with IC_50_ = 50 nM and 500 nM respectively [83], [84]. At 10 µM the residual activity (% of control) was found to be 2±1, 10±1, 96±2, and 93±4 for the isoforms α to δ, respectively [83]. Therefore, the inhibitory effect occurs mostly over the isoform α and to a lesser extends over the isoform β. SB203580 also inhibited the serum withdrawal effects on COX-2 mRNA in MDA-MB-231 cells with IC_50_ ∼ 10 µM, a value which was higher than in monocytic cells and synoviocytes [85]. Therefore, we used SB203580 at concentrations ranging 1–20 µM.

**Figure 6:**
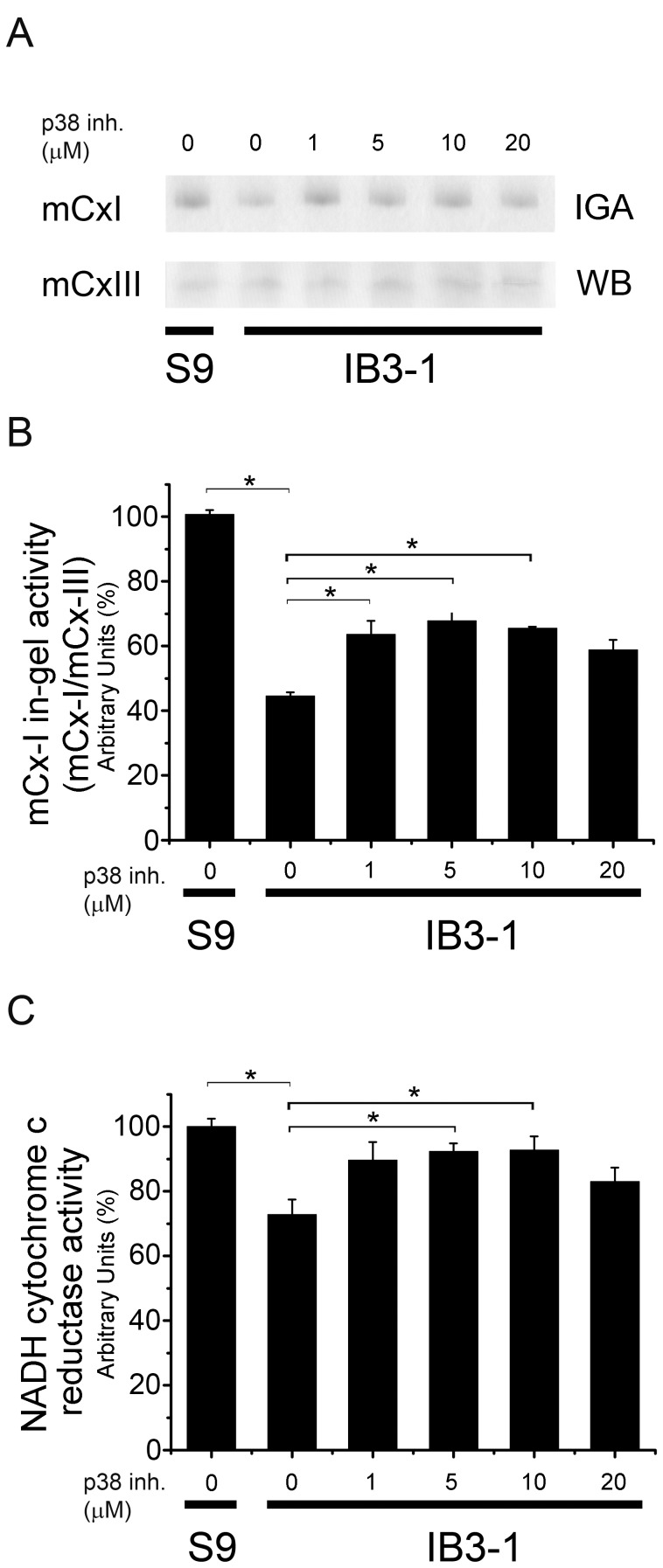
P38/MAPK1 inhibition. After 24-1 cells were incubated for 24 h with increasing concentrations of the p38 MAPK inhibitor SB203580 (1, 5, 10 and 20 µM) and mCx-I activity was measured by using BN-PAGE and spectrophotometry. A: mCx-I in-gel activity (IGA) and mCx-III (WB). B: Densitometric quantification and statistical analysis of the results shown in panel A. IGA of mCx-I was calculated as the ratio mCx-I (IGA)/mCx-III (WB). C: Spectrophotometric measurements of the mitochondrial NADH-cytochrome c reductase activity in the same experiments of panel A, expressed as percentage (%) relative to S9 control values. Measurements were performed in triplicate and data were expressed as mean ± SE of three independent experiments (n = 3). *indicates p<0.05 compared with basal IB3-1 cells.

Contrary to the results shown above for MEK1/2 and JNK inhibitors, the p38 SB203580 inhibitor was able to revert the low mCx-I activity of IB3-1 cells. As shown in Figure 6, maximal response to SB203580 was obtained at ∼5 µM (24 h incubation). Using BN-PAGE (Fig. 6A and 6B), the p38 inhibitor was able to recover near 70% of the activity measured in S9 cells. On the other hand, by using spectrophotometric measurements of the mitochondrial NADH-cytochrome c reductase activity (Fig. 6C), in the presence of 5 µM of inhibitor, the IB3-1 cells reached over 90% of the activity measured in S9 cells. Thus, near a full recovery of the mCx-I activity of IB3-1 cells was obtained through p38 inhibition.

### Effects of NF-κB Inhibition on mCx-I Activity of S9 and IB3-1 Cells

**Figure 7:**
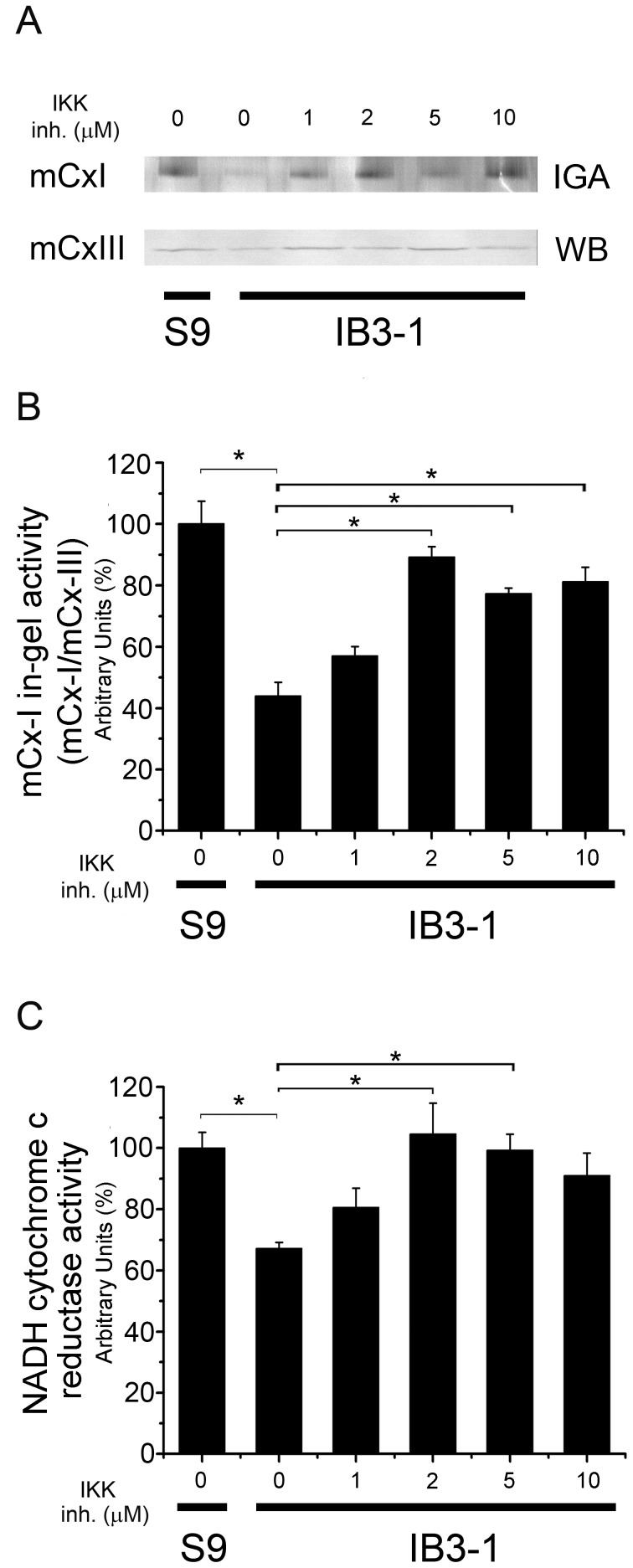
NF-κB pathway inhibition. S9 and IB3-1 cells were preincubated for 24 h in serum-free media and then the IB3-1 cells were incubated for additional 24 h in the presence of increasing concentrations of IKK inhibitor III (1, 2, 5 and 10 µM). A: mCx-I in-gel activity (IGA) and mCx-III (WB). B: Densitometric quantification and statistical analysis of the results shown in panel A. IGA of mCx-I was calculated as the ratio mCx-I IGA/mCx-III WB. C: Spectrophotometric measurements of the mitochondrial NADH-cytochrome c reductase activity in the same experiments of panel A, expressed as percentage (%) relative to S9 control values. Measurements were performed in triplicate and data were expressed as mean ± SE of three independent experiments (n = 3). *indicates p<0.05 compared with basal IB3-1 cells.

As IL-1β exerts many of its biological effects by activating the transcription factor nuclear factor-kappa B (NF-κB) [15], [18], [86], we explored whether the inhibition of this signaling pathway can also help to recover the mCx-I activity of IB3-1 cells. For this purpose we used the IKK inhibitor III/BMS-345541, which inhibit IKK (IκB kinase). It has IKK-2 IC_50_ ∼ 0.3 µM and IKK-1 IC_50_ ∼ 4 µM [87], and inhibited the stimulated phosphorylation of IκB in THP-1 in cells with IC_50_ ∼ 4 µM [87]. Therefore, we used this inhibitor at concentrations ranging (1–10 µM). As shown in Figure 7 treatment of IB3-1 cells with IKK inhibitor III led to a significant (p<0.05) increase on the mCx-I in-gel activity (IGA) as compared to untreated control cells. Noteworthy, by using spectrophotometry (Figure 7C), a complete recovery of the mitochondrial NADH-cytochrome c reductase activity of IB3-1 cells was observed in the presence of IKK inhibitor III (compared to S9 cells).

**Figure 8:**
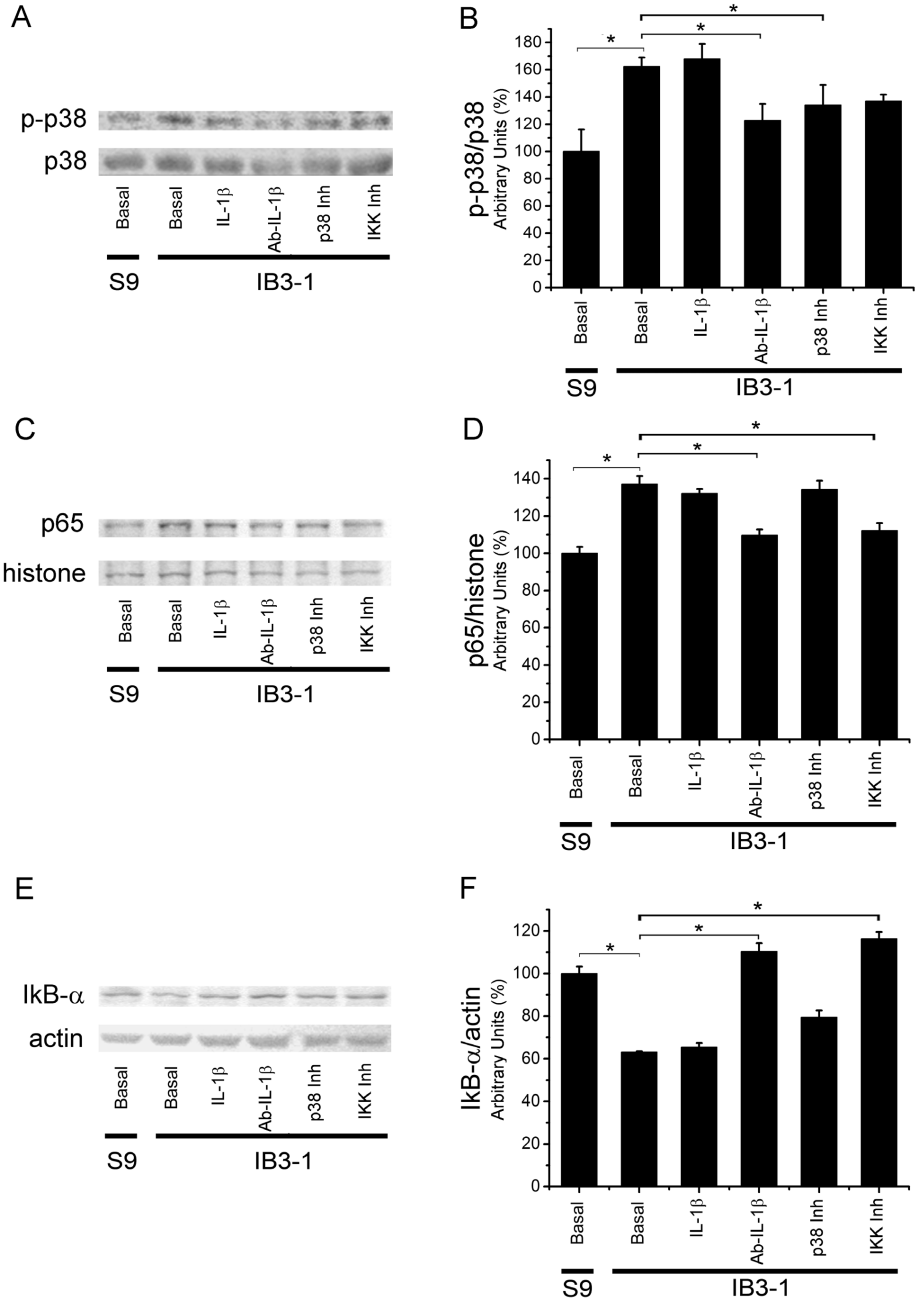
P38 and NF-κB activation in S9 and IB3-1 cells. Cells were preincubated 24-1β (5ng/ml), anti-IL-1β blocking antibody (Ab-IL-1β) (30 ng/ml), p38 inhibitor SB203580 (p38 Inh) (5 µM), or IKK inhibitor III (IKK Inh) (2 µM), for another 24 h in serum-free media. A: Representative WB of phospho-p38 (p-p38) and total p38 of whole cellular lysates from S9 an IB3-1 cells. B: Densitometric quantification and statistical analysis of p38 phosphorylation status (calculated as the p-p38/p38 ratios for each experimental condition). C: Representative WB of NF-κB p65 and histone H1 from nuclear extracts of S9 and IB3-1 cells. D: Densitometric quantification and statistical analysis of the results shown in panel C (calculated as p65/histone ratio for each experimental condition). E: Representative WB of IκB-α and actin of whole cellular lysates from S9 and IB3-1 cells. F: Densitometric quantification and statistical analysis of the results shown in panel C (calculated as IκB-α/actin ratio for each experimental condition). The results were expressed as percentage (%) relative to S9 control values. Measurements were performed in triplicate and data are expressed as mean ± SE of three independent experiments (n = 3). *indicates p<0.05 compared to basal IB3-1 cells.

To make sure that p38 was activated in IB3-1 cells, the phosphorylation status of p38 (p-p38) was tested by Western blots. As shown in Figure 8A (WB) and 8B (quantification), the phosphorylation of p38 was higher in IB3-1 CF cells than in S9 cells. The phosphorylation of p38 in IB3-1 cells stimulated with IL-1β (5 ng/ml) was unaltered as compared to untreated IB3-1 cells, suggesting again that the autocrine IL-1β stimulus is enough to saturate its signaling pathway. However, the phosphorylation of p38 in IB3-1 cells treated with anti-IL-1β antibody or p38 inhibitor was significantly (p<0.05) decreased compared to IB3-1 untreated cells. The IKK inhibitor III also showed some reduction on the p-p38 levels, although the differences were not significant compared to basal IB3-1 p-p38 levels. Altogether, these results reinforce the idea that autocrine IL-1β is responsible for the reduced mCx-I activity observed in IB3-1 cells and suggest a possible role of p38 MAPK in the autocrine effects of IL-1β over the mCx-I activity.

To confirm the activation of NF-κB in IB3-1 cells, the relative amount of p65 were measured by using Western blots of nuclear extracts. As shown in Figure 8C and D, the amount of nuclear p65 was significantly increased (p<0.05) in basal IB3-1 cells compared to S9 cells. This p65 levels were maintained in IB3-1 cells treated with IL-1β (signal already saturated) or p38 inhibitor (no effect). In contrast, as expected, p65 nuclear translocation in IB3-1 cells treated with anti-IL-1β antibody or IKK inhibitor III was decreased (p<0.05) compared to IB3-1 basal cells. Finally, cytoplasmic IκBα showed the opposed behavior to p65, as expected for NF-κB activation (Fig. 8E and F).

The results obtained with IKK inhibitor III, as those obtained with the p38 inhibitor SB203580, or the IL1 β blocking Ab and IL1RN, also reinforce the idea that autocrine IL-1β is responsible for the reduction of mCx-I observed in IB3-1 cells and suggest that the NF-kB also has a role in the IL-1β effects on the reduced mCx-I activity of IB3-1 cells.

### Mitochondrial and Cellular ROS Levels on S9 and IB3-1 Cells

**Figure 9:**
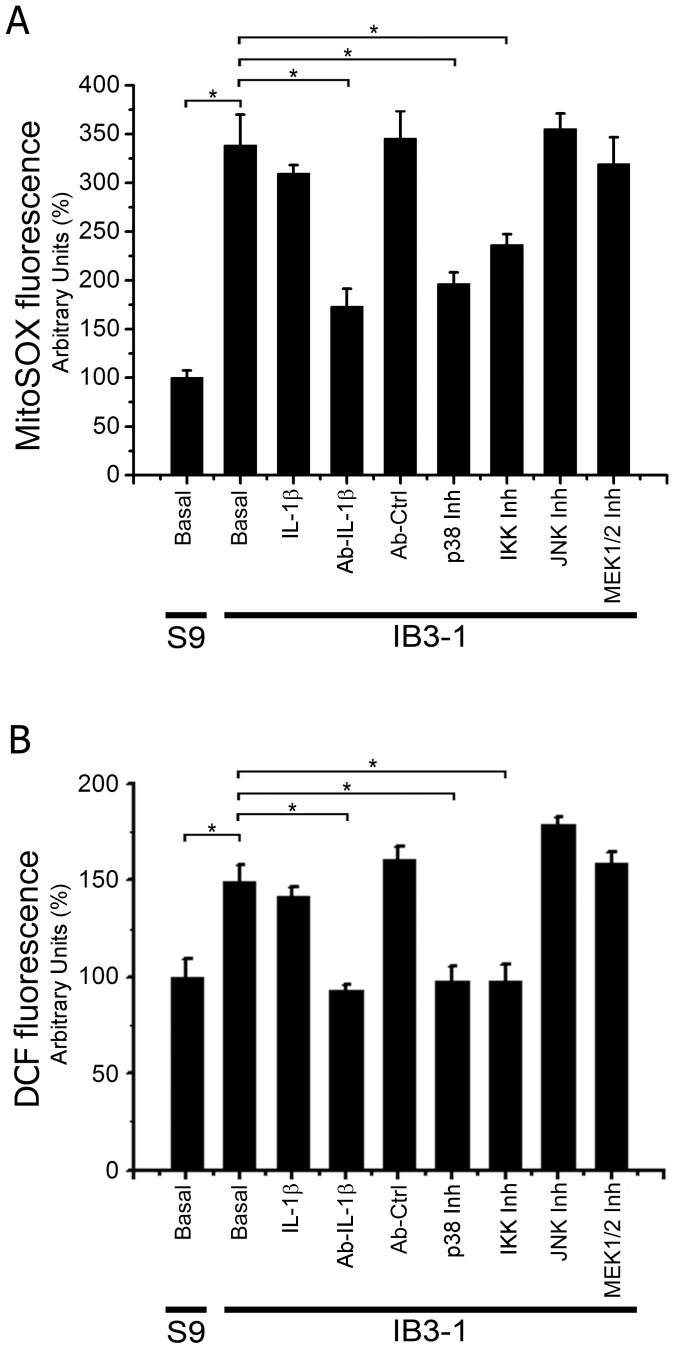
ROS levels in IB3-1 and S9 cells. S9 and IB3-1 cells were preincubated 24 h in serum free media. Then, the media was replaced (serum free, in the presence of a CFTR-stimulating cocktail) and the cells incubated another 24 h. The IB3-1 cells were treated in this second incubation as indicated. IL-1β: IL-1β (5 ng/ml); Ab-IL-1β: anti-IL-1β blocking antibody (30 ng/ml); Ab-Ctrl: anti-Histone H1 monoclonal antibody as negative control for antibody incubation (30 ng/ml); p38 Inh: p38 inhibitor SB203580 (5 µM); IKK Inh: IKK inhibitor III (2 µM); JNK Inh: JNK inhibitor SP600125 (5 µM); MEK1/2 Inh: MAPK/AP-1 pathway inhibitor U0126 (5 µM). A: MitoSOX fluorescence (5 µM for 10 min in Hank’s buffer). B: DCF fluorescence (10 µM DCFH-DA for 40 min in Hank’s buffer); Ab-Ctrl: anti-JNK2 monoclonal antibody as negative control. Results were expressed as percentage (%) relative to S9 control values. Measurements were performed in triplicate and data are expressed as mean ± SE of three independent experiments (n = 3). *indicates p<0.05 compared to IB3-1 cells.

It has been reported that inhibition of mCx-I might increase ROS levels in certain cells and conditions [88], [89]. Therefore, we speculated that autocrine effects of IL-1β might also be responsible for the increased ROS levels observed by other authors in IB3-1 cells [33]. As shown in Figure 9A, the mitochondrial ROS levels, measured by using MitoSOX, were found significantly increased (p<0.05) in IB3-1 cells compared to S9 cells, reflecting an increased oxidative stress. Exogenously added IL-1β was not able to further increase ROS levels in IB3-1 cells, which again suggests that the IL-1β pathway is already saturated in these cells, as it was observed measuring the mCx-I activity. In addition, the anti-IL-1β blocking monoclonal antibody was able to reduce ROS levels of IB3-1 cells to near 50%. The IKK inhibitor III (2 µM) or the MAPK1/p38 inhibitor, SB203580, (5 µM) had both a significant inhibitory effect on the ROS levels of IB3-1 cells. On the other hand, also as occurred with the inhibition of mCx-I activity, the inhibitor of MEK1/2, U0126 (5 µM) and of JNKs, SP600125 (5 µM), were not able to modify mitochondrial ROS levels in IB3-1 cells. Similar results were found when the cellular ROS levels were measured by using the fluorescent probe DCFH-DA, as shown in Figure 9B.

### Effects of IL-1β and its Inhibitors on mCx-I Activity and ROS Levels of Caco-2 Cells Transfected with shRNA Specific for CFTR

**Figure 10:**
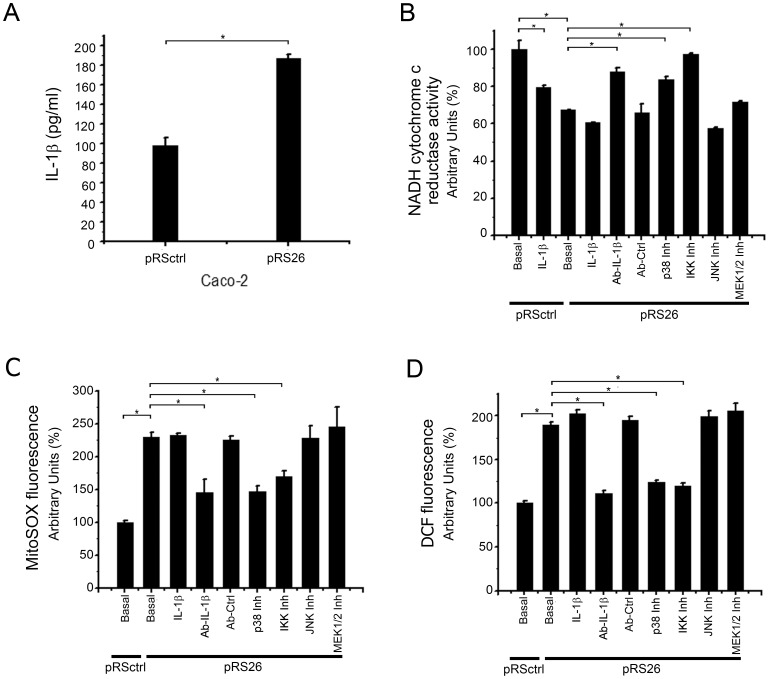
Il-1β secretion, Mitochondrial NADH cytochrome c reductase activity and ROS levels in stable CFTR knock down cells. Caco-2/pRSctrl cells (transfected with pRS control) and Caco-2/pRS26 cells (transfected with the shRNA pRS26) were preincubated 24 h in serum free media. A: Immune-dot blotting quantification of the IL-1β present in culture media. The results were expressed as pg/ml. B: Cells were treated for additional 24 h, as indicated. IL-1β: IL-1β (5 ng/ml); Ab-IL-1β: anti-IL-1β blocking antibody (30 ng/ml); Ab-Ctrl: anti-Histone H1 monoclonal antibody as negative control (30 ng/ml); p38 Inh: p38 inhibitor SB203580 (5 µM); IKK Inh: IKK inhibitor III (2 µM); JNK Inh: JNK inhibitor SP600125 (5 µM); MEK1/2 Inh: MAPK/AP-1 pathway inhibitor U0126 (5 µM). Spectrophotometric measurements of the mitochondrial NADH-cytochrome c reductase activity, expressed as percentage (%) relative to Caco-2/pRS26 control values. C: Mitochondrial ROS levels in the same experiments of panel B; the figure shows the MitoSOX fluorescence (5 µM for 10 min in Hank’s buffer). Results were expressed as percentage (%) relative to Caco-2/pRSctrl control values. D: Cellular ROS levels in the same experiments of panel B; the figure shows the DCF fluorescence (DCFH-DA 10 µM for 40 min in Hank’s buffer). Results were expressed as percentage (%) relative to Caco-2/pRSctrl control values. Measurements were performed in triplicate and data are expressed as mean ± SE of two independent experiments (n = 2). *indicates p<0.05 compared to Caco-2/pRS26 cells.

To determine if the overexpression of IL-1β and the reduction on the mCx-I activity observed in IB3-1 could also be observed in a different cell system, we then used Caco-2 cells (which express wt-CFTR) transfected with a control plasmid pRSctrl or with a plasmid pRS26 containing a CFTR-specific shRNA. These stable transfected cells were selected, cloned and characterized in a previous work [47]. As shown in Figure 10A, secreted IL-1β was significantly increased (∼ 190%) in Caco-2/pRS26 cells (187±4 ng/ml) compared to Caco-2/pRSctrl control cells (98±8 ng/ml). The secretion rate was 1.6 fmol/10^6^cells/h (13.9 fg/ml/mg/h) and 0.8 fmol/10^6^cells/h (6.6 fg/ml/mg/h), respectively. In addition, as shown in Figure 10B, the CFTR shRNA induced a significant reduction of NADH cytochrome c reductase activity in Caco-2/pRS26 cells compared to Caco-2/pRSctrl control cells. The values were similar to those previously reported for these stably-transfected cells [47].

When the Caco-2/pRSctrl control cells and Caco-2/pRS26 cells were incubated with 5 ng/ml of IL-1β, for 24 h in serum-free medium, the mitochondrial NADH-cytochrome c reductase activity in Caco-2/pRSctrl cells was significantly reduced (p<0.05) compared to untreated Caco-2/pRSctrl cells. No significant differences were observed between IL-1β treated and untreated Caco-2/pRS26 cells, again in agreement with the idea that autocrine IL-1β secretion was enough to saturate its own signaling. As shown in Figure 10B, the IL-1β blocking antibody significantly increased (p<0.05) the mitochondrial NADH-cytochrome c reductase activity of Caco-2/pRS26 compared to the untreated cells. Treatments of Caco-2/pRS26 cells with the IKK inhibitor III (2 µM) or the p38 MAPKs inhibitor SB203580 (5 µM) led to a significant increase on the mitochondrial NADH-cytochrome c reductase activity as compared with untreated cells. In addition, MEK1/2 and JNK inhibitors were ineffective in reversing the reduced mitochondrial NADH-cytochrome c reductase activity. These results are all in agreement with those obtained when the same experiments were done by using IB3-1 and S9 cells (Fig. 2–7).

We next studied the mitochondrial ROS levels in Caco-2/pRSctrl (control cells) and CFTR knock-down Caco-2/pRS26 cells. As shown in Figure 10C, the mitochondrial ROS levels, measured by using the mitochondrial ROS probe MitoSOX, were found significantly increased in Caco-2/pRS26 cells compared to Caco-2/pRSctrl control cells. Again, as occurred with IB3-1 cells, exogenously added IL-1β was not able to further increase ROS levels in Caco-2/pRS26 cells. Anti-IL-1β blocking monoclonal antibody was able to significantly (p<0.05) reduce ROS levels of Caco-2/pRS26 cells. Finally, the IKK inhibitor III (2 µM) and the MAPK1/p38 inhibitor (SB203580) (5 µM) have a strong inhibitory effect on ROS levels of Caco-2/pRS26 cells. Once more, the inhibitors of MAPKK/MEK1/2 (U0126, 5 µM) and JNK II (SP600125, 5 µM), were not able to modify ROS levels in Caco-2/pRS26 cells. Similar results were found when the cellular ROS levels were measured by using the fluorescent probe DCFH-DA, as shown in Figure 10D. However, the contribution of other sources of ROS levels, beside mitochondria, such as the DUOX1/2 system, cannot be ruled-out [90]. On the other hand, the higher reduction observed in the cellular ROS (cROS) levels compared to the mitochondrial ROS (mROS) levels in the presence of the IL-1β blocking Ab might be in part due to effects of the scavenger systems of cytoplasm, including glutathione, peroxiredoxin-6 and superoxide dismutase [35], and not only due to a regulation in the production of cROS and mROS. Taken together, all the results obtained in Caco-2 cells are in agreement with those obtained with IB3-1 cells. In conclusion, the inhibitors of p38 MAPKs and IKKs, or the IL-1β blocking Ab and the IL1RN, were able to revert the mCx-I activity and significantly reduce the ROS levels in cells with impaired CFTR activity (IB3-1 and Caco-2/pRS26 cells).

## Discussion

The aim of this work was to test the hypothesis that the reduced mCx-I activity observed in CF cells or in cells with impaired CFTR activity [35], [47] could be at least partially attributed to an autocrine effect [91], [92] of IL-1β.

After verifying that in our culture conditions the IB3-1 cells overexpressed IL-1β and had a reduced mCx-I activity, we explored whether the IL-1β secreted by IB3-1 cells was responsible for the mCx-I inhibition. For this purpose, the IB3-1 cells were incubated in the presence of an anti-IL-1β monoclonal antibody, which blocks the biological activity of IL-1β [93]. Noteworthy, in the presence of blocking antibody, a significant and almost full recovery of the mCx-I activity was observed for IB3-1 cells, with values approaching those of S9 cells. Similar effects were observed by using the interleukin-1 receptor antagonist peptide IL1RN [94], [95]. Thus, an autocrine [91], [92] effect of IL1-β appears to be responsible for the reduced mCx-I activity observed in IB3-1 cells. Further evidence in support of this hypothesis was obtained by using pharmacological inhibitors of different branches of the IL-1β signaling pathway. The inhibitors U0126 (MEK1/2) and SP600125 (JNKs) were not able to recover the mCx-I activity of IB3-1 cells. Since U0126 blocks AP-1 signaling through MEK1/2 inhibition [79] and SP600125 blocks c-Jun phosphorylation and activation of AP-1 through inhibition of JNKs, these results suggest that the AP-1 pathway is not involved in the reduction of the mCx-I activity observed in IB3-1 CF cells. Noteworthy, the p38 MAPK inhibitor SB203580 was able to induce a significant recovery in the mCx-I activity of IB3-1 cells. The effect was even more pronounced when the IKK inhibitor III was used, which was able to fully recover the mCx-I activity of IB3-1 cells compared to S9 cells.

Regarding the levels of reactive oxygen species (ROS), it has been reported that under certain conditions a reduced mCx-I activity might lead to increased levels of mitochondrial (mROS) [96]. In addition, Velsor et al. [33] found increased mitochondrial (and cytoplasmic) ROS levels in IB3-1 CF cells (these mitochondrial effects in CF were reviewed in [35]). Therefore, we measure the mitochondrial and cellular ROS levels of IB3-1 cells treated or not with IL-1β blocking mAb and IL-1β pathway inhibitors. We found elevated mitochondrial and cellular ROS levels in IB3-1 cells compared to S9 cells. Similarly to the rescued effects on mCx-I activity, the anti-IL-1β blocking antibody was able to decrease mitochondrial ROS levels of IB3-1 cells over 50% and the cellular ROS levels near 100%. Addition of IL-1β (5 ng/ml) to IB3-1 cells could not further increase the ROS levels, suggesting again (as observed for the mCx-I activity) that the IL-1β signaling was already saturated by the endogenous autocrine secretion of IL-1β. On the other hand, the inhibitor SB203580 (p38 MAPK) and the IKK inhibitor III (NF-κB pathway), were both able to produce a significant reduction of mitochondrial ROS levels in IB3-1 cells (∼ 30 and 40% respectively) and a reduction to near basal values in the cellular ROS levels. These results correlate with the effects of these inhibitors on mCx-I activity. In addition, as occurred with the mCx-I activity, the AP-1 pathway inhibitors (U0126 and SP600125) showed not significant differences compared to control IB3-1 cells, suggesting that the AP-1 signaling pathway is not involved in the increased ROS levels found in IB3-1 CF cells.

To confirm the results obtained in IB3-1 we developed a different cellular model, made by using Caco-2 coloncarcinoma cells. As expected, Caco-2/pRS26 CFTR-shRNA cells showed a significant reduction in the mitochondrial NADH-cytochrome c reductase activity compared to Caco-2/pRSctrl control cells. Incubations with IL-1β (5 ng/ml) resulted in a significant reduction of mitochondrial NADH-cytochrome c reductase activity only in Caco-2/pRSctrl control cells (a small but not significant reduction was observed in Caco-2/pRS26). In addition, the reduced mCx-I activity of Caco-2/pRS26 cells was recovered by incubation with the IL-1β blocking mAb and a significant recovery of the mitochondrial NADH-cytochrome c reductase activity was observed for Caco-2/pRS26 cells in the presence of IKK inhibitor III or the p38 MAPK inhibitor SB203580. Also, elevated ROS levels were found in Caco-2/pRS26 cells compared to Caco-2/pRSctrl control cells (similar preliminary results were previously obtained with Caco-2/pRS25 cells [97]). Finally, the anti-IL-1β blocking antibody decreased ROS levels in this CFTR impaired cells. Moreover, the IKK inhibitor III and the p38 inhibitor were also able to decrease the ROS levels in Caco-2/pRS26 cells.

Thus, the results obtained measuring mitochondrial ROS levels are in agreement with those obtained by using IB3-1 cells, and confirm the presence of autocrine IL-1β affecting the mCx-I activity and the ROS levels of cells with impaired CFTR activity (IB3-1 and Caco-2/pRS26 cells). The alternative possibility of the absence or reduction of IL-1β receptor levels or signaling in IB3-1 cells should not be ruled-out, although it seems unlikely. In fact, the IL-1β blocking Ab was able to reduce the cellular ROS levels near basal values, suggesting that the IL-1β receptor signaling is not affected in IB3-1 cells (since in the absence of IL-1β blocking Ab or IL1RN the mCx-I activity and ROS levels are affected by the secreted IL-1β). Regarding the cellular ROS levels, in addition to the ROS produced by mitochondria, other sources of ROS such as the DUOX1/2 system cannot be ruled-out [90]. Since the recovery obtained in ROS levels using the blocking Ab or the inhibitors was not total, other mechanisms of ROS generation might also be contributing to the total ROS levels. We do not known yet whether the high ROS levels affect the mCx-I activity or vice versa, the high ROS levels are a consequence of the reduced mCx-I activity. Probably both possibilities are operating simultaneously, since it has been shown that a reduced mCx-activity can induce ROS generation [88], [89] and also, the generation of ROS can reduce the mCx-I activity, since it is highly susceptible to oxidation [98], [99]. In addition, an alternative scenario for the observed effects on ROS and IL-1β is one in which CFTR is affecting first the ROS levels, increasing in turn NF-κB activity and then IL-1β levels (CFTR failure → ROS → NF-kB → IL-1β). However, it seems also unlikely, since the stimulation of ROS would be upstream of IL-1β and in such case the blocking Ab should not be able to reduce the ROS levels, as we have observed here (unless this alternative mechanisms is accounting for the remaining mitochondrial ROS levels observed in the presence of the blocking Ab). Further studies are needed to determine which is first, ROS, IL-1β or both in parallel. Our present data rather support the first hypothesis (CFTR → IL-1β→ ROS), although the data are not conclusive yet.

**Figure 11:**
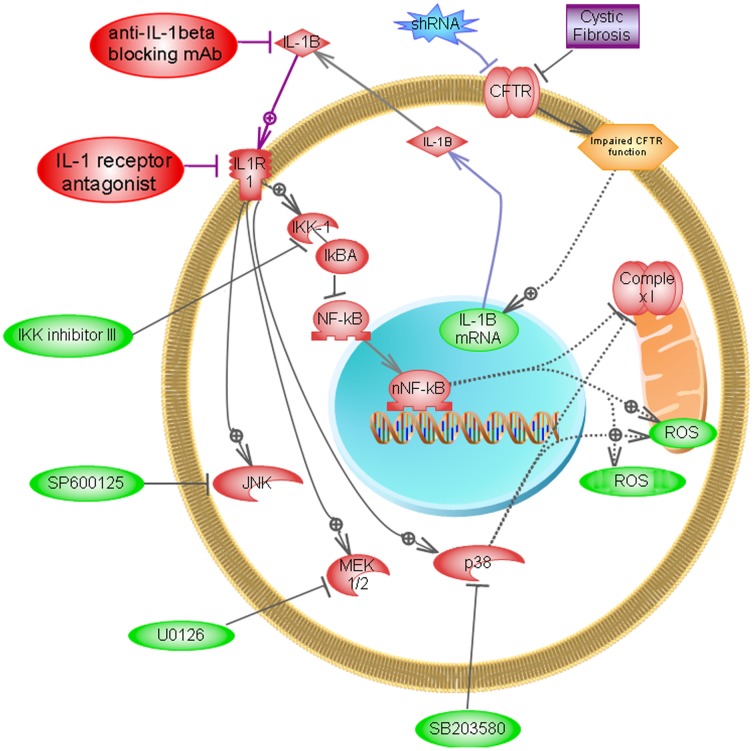
Graphical summary for IL-1β effects on mCx-I activity and mitochondrial ROS levels in IB3-1 and Caco-2/pRS26. The figure illustrates the interactions among the different proteins, kinases or small molecules involved in this work. The interactions were drawn by using the software Pathway Studio (v 9, Elsevier). Arrows with the+symbol represent stimulations and those with the -| symbol represent inhibition. Green ellipses: small molecules; red sickle-vertex: kinases; purple rectangle: disease (CF); blue star-vertex: shRNA specific for CFTR. The results obtained with IL-1β blocking Ab or with the receptor inhibitor IL1RN suggest that an autocrine IL-1β signaling is responsible for the reduced mCx-I activity and the increased ROS levels seen in IB3-1 CF cells or in Caco-2/pRS26 cells. Inhibition of NF-κB or p38 MAPK also resulted in increased mCx-I activity and decreased ROS levels. The inhibition of MEK1/2 or JNKs (AP-1 pathway) had no effects. The mechanisms by which CFTR increases IL-1β and IL1-β, p38 MAPKs or NF-κB inhibit mCx-I and increase ROS levels remain to be determined (dotted lines).

A graphical summary of the results obtained here is shown in Figure 11, made by using the Pathway Studio software v.9 [100]. The results imply that autocrine IL-1β has a major role in the reduction of the mCx-I activity observed in cultured CF cells or cells with impaired CFTR activity (IB3-1, Caco-2/pRS26 cells). Interestingly, the IKK inhibitor III, the IL-1β blocking Ab or the IL-1RN, were able to fully restore the mCx-I activity, and to produce a significant reduction in the ROS levels, to near basal values for cellular ROS levels. In consequence, IL-1β blocking reagents and inhibitors of NF-κB or p38 MAPKs might have therapeutic significance for CF patients. Since the IL-1β autocrine effects over mCx-I and ROS were observed in cell cultures in the absence of bacterial infections, the IL-1β autocrine effects appear to be a primary, intrinsic characteristic of CF cells. However, it should be pointed out that the results obtained here with IB3-1 and Caco-2/pRS26 cells should not be generalized without caution. In fact, there is no consensus regarding whether or not CF cells significantly differ from normal with respect to ROS, NF-κB activity, and IL1β expression or signaling [27], . Finally, the physiological significance of the reduced mCx-I activity and the increased ROS levels observed for IB3-1 and Caco-2/pRS26 cells is yet unknown.
